# Frailty and cardiovascular safety of JAK inhibitors versus TNF inhibitors in rheumatoid arthritis: a real-world comparative study of drug effects and patient profiles

**DOI:** 10.3389/fphar.2025.1565909

**Published:** 2025-04-25

**Authors:** Ettore Silvagni, Alessandra Bortoluzzi, Giuseppe Occhino, Giuseppe Bellelli, Carlo Garaffoni, Paolo Delvino, Olivia Leoni, Maria Grazia Valsecchi, Carlo Alberto Scirè, Paola Rebora

**Affiliations:** ^1^ Rheumatology Unit, Department of Medical Sciences, University of Ferrara and Azienda Ospedaliero-Universitaria S. Anna, Cona (Ferrara), Italy; ^2^ Bicocca Bioinformatics, Biostatistics and Bioimaging Centre - B4 School of Medicine, University of Milano-Bicocca, Milan, Italy; ^3^ Acute Geriatric Unit, IRCCS San Gerardo dei Tintori Foundation, Monza, Italy; ^4^ School of Medicine and Surgery, University Milano-Bicocca, Milan, Italy; ^5^ Rheumatology Unit, IRCCS San Gerardo dei Tintori Foundation, Monza, Italy; ^6^ General Directorate for Health, Milan, Lombardy, Italy; ^7^ Unit of Clinical Epidemiology and Biostatistics, IRCCS San Gerardo dei Tintori Foundation, Monza, Italy

**Keywords:** rheumatoid arthritis, frailty, JAK inhibitors, TNF inhibitors, cardiovascular events, healthcare administrative databases

## Abstract

**Objective:**

The aim of this study was to comparatively assess the risk of cardiovascular events (CVEs) in rheumatoid arthritis (RA) patients treated with Janus kinase inhibitors (JAKis) or tumor necrosis factor inhibitors (TNFis) and to explore the interactions with patient profiles [including age, baseline cardiovascular (CV) risk, and frailty, which is a state of decreased physiological reserve, assessed using a validated frailty index (FI) for healthcare administrative databases (AHDs)].

**Methods:**

This retrospective study was based on AHDs of the Lombardy region, Italy (from 1st January 2020 to 31st December 2023). Cox regression models, both crude and adjusted, were applied to estimate the association between treatments and outcomes [CVEs, major adverse cardiovascular events (MACEs), and thromboembolic events (TEs)]. We tested the interaction between the drug treatment and the regulatory agency prescription rule changes [before or after 06th July 2021, the date of the first European Medicine Association (EMA) pronouncement on tofacitinib safety] or patient profiles.

**Results:**

We identified 7,541 therapeutic courses in 5,563 patients: 2,343 started as TNFi users, 1,443 as JAKi users, and 1,777 started with other drugs (1,459 days of follow-up). The crude incidence rates (IRs) for new CVEs were 16.6 [95% confidence intervals (95% CI): 12.8–21.2] and 18.6 (95% CI: 14.2–23.9) per 1,000 person-years (PYs) for TNFi and JAKi users, respectively. Exposure to JAKis was not associated with a significantly increased risk of CVEs [adjusted hazard ratio (HR): 0.92; 95% CI: 0.64–1.32], MACEs (adjusted HR: 0.71; 95% CI: 0.37–1.33), or TEs (adjusted HR: 1.53; 95% CI: 0.65–3.65) compared to TNFis. Each 0.1-point increment of the FI significantly increased the HR for new CVEs (HR: 1.80; 95% CI: 1.48–2.19), MACEs (HR: 1.66; 95% CI: 1.10–2.51), and TEs (HR: 1.69; 95% CI: 1.03–2.78). When assessing the interaction between the period of drug delivery and the treatment with JAKis on the risk of new CVEs, no significant interaction was observed (p = 0.838), while the interaction was statistically significant for baseline CV risk (p = 0.007).

**Conclusion:**

RA patients treated with JAKis in real-world settings have a risk of developing CVEs no higher than those of TNFi users, but potential signals remain for TEs, even if the sample was not sufficiently powered. Patient profiles, particularly the frailty, have a more substantial impact on the risk of CVEs than the specific disease-modifying anti-rheumatic drug (DMARD) choice.

## 1 Introduction

Janus kinase (JAK) inhibitors (JAKis) serve as important therapeutic options in the management of rheumatoid arthritis (RA) (Smolen et al., 2023). However, it is crucial to thoroughly consider relevant risk factors when contemplating the use of targeted synthetic disease-modifying anti-rheumatic drugs (ts-DMARDs), like JAKis. These remarks stem from the findings of the ORAL Surveillance trial, which raised concerns about the cardiovascular (CV) safety of tofacitinib compared to that of tumor necrosis factor inhibitors (TNFis) in RA patients aged 50 years or older with at least one additional CV risk factor (Ytterberg et al., 2022). The implications regarding the CV safety profile of various JAKis led the U.S. Food and Drug Administration (FDA) to issue a boxed warning regarding the increased risk of blood clots and death associated with the use of tofacitinib, ratified by the European Medicine Association (EMA) on 06th July 2021. More recently, on 23rd January 2023, the EMA human medicine committee endorsed measures recommended by the Pharmacovigilance Risk Assessment Committee (PRAC) to minimize the risk of serious side effects, including CV events (CVEs) and blood clots, with all the JAKis approved for RA (EMA, 2022). The CV safety assessment is a crucial matter, and the research agenda of the European Alliance of Associations for Rheumatology (EULAR) has specifically addressed the question of whether the cardiovascular and malignancy risks associated with JAKis observed in the ORAL Surveillance study differ between JAK-1 or JAK1/2-selective agents *versus* pan-JAK is (Smolen et al., 2023), underscoring the need to scrutinize the safety profiles of individual JAKis in relation to their diverse selectivity.

In the last years, several real-life studies addressed the matter of CV safety of JAKis out of the shadow of clinical trials, with a number of reports depicting a different scenario from what emerged in the ORAL Surveillance, suggesting that the CV risk linked to RA DMARDs is much more complex than a pure class effect (Garaffoni et al., 2022; Khosrow-Khavar et al., 2022; Frisell et al., 2023; Molteni et al., 2024). The analysis of register-based cohorts and healthcare administrative databases (AHDs) reported few differences in terms of cardiovascular events between the treatments, suggesting that the presence of selected patient profiles or individual risk factors might overcome the effects of the hypothesized drug class (Bower et al., 2023; Frisell et al., 2023). To this end, a relatively innovative concept in rheumatology which more accurately captures the clinical heterogeneity of individuals with similar risk factors than chronological age and comorbidities is frailty (Rockwood and Mitnitski, 2007; Bellelli et al., 2023; Bellelli et al., 2024). Frailty is defined as a dynamic state of decreased physiological reserves with a compromised capacity to maintain homeostasis because of time-related deficit accrual, and it quantitatively summarizes the vulnerability. Its presence in patients with rheumatic musculoskeletal diseases is common, despite being under-recognized and under-reported in both clinical trials and observational studies (Salaffi et al., 2019; 2023; Motta et al., 2020; Gao et al., 2022). It is conceivable that frailty can contribute to the CV safety aspects of DMARDs in RA, in line with other individual risk factors and, consequently, to the regulatory agencies’ warning releases (Corrao et al., 2024).

To further elucidate the CV safety profile of JAKis with respect to patient profiles, we conducted this study with the main objective to estimate and compare the risk of CVEs in patients with RA exposed to JAKis *versus* TNFis within a real-world population based on AHDs. Secondary objectives were to estimate (i) the risk of different types of CVEs [major adverse cardiovascular events (MACEs) and thromboembolic events (TEs)], (ii) the influence of single JAKis on the risk of CVEs, and (iii) the influence of the regulatory agencies’ warnings conditioning the prescription attitudes or the patient profiles (including age, baseline CV risk, and frailty) on the overall CVE risk. Specifically, we assessed how a validated electronic-regional healthcare database frailty index (e-RHD-FI) modified the risk of CVEs, MACEs, and TEs in RA exposed to JAKis *versus* TNFis, as well as the interaction between the drug treatment and frailty on the overall CVE risk.

## 2 Materials and methods

### 2.1 Data source

This retrospective cohort study was performed taking advantage of AHDs, and it was conducted following the RECORD statement checklist (Supplementary Material) (Benchimol et al., 2015).

The source registry was an electronic database that contains fields built as an obligatory menu, limiting the possible errors and missing data. Individuals aged ≥18 years who were beneficiaries of the Lombardy Regional Health System on 31st December 2019 (n = 8,496,045) were selected for the analyses. This study was approved by the Brianza Institutional Review Board (3356-07/08/2020), and it was performed in accordance with the ethical standards as laid down in the 1964 Declaration of Helsinki and its later amendments.

### 2.2 Participants

The study population included RA patients, identified through diagnosis codes (ICD9-CM 714.0) on 1st January 2020 or thereafter, and starting their exposure to TNFis, JAKis, or non-TNFis/non-JAKis [interleukin-6 inhibitors (IL-6i), rituximab (RTX), and abatacept (ABT)] during the period of interest from 1st January 2020 to 31st December 2023, both in first-line and subsequent therapeutic lines. For each patient, frailty was assessed according to the e-RHD-FI, defined by Rebora et al. (2023).

### 2.3 Demographic and clinical variables collected

For each patient, a core set of data was included: demographics (birth date, gender, and death), drug delivery [Anatomical Therapeutic Chemical (ATC)] codes, date of drug delivery, quantity, date of embarkment, diagnosis codes, date of diagnosis code release, outpatient services ICD-CM [International Classification of Diseases, 9th revision, Clinical Modification (ICD-9-CM) expanded codes and dates], hospital discharge forms with information on hospitalization beginning and ending dates, diagnoses and procedures (ICD-9-CM), and disease-related group (DRG 24).

### 2.4 Drug exposure and time-dependent covariates

We considered the exposure to a specific treatment as the time from the first drug delivery in the period of the study to the end of the coverage of the last consecutive delivery plus 6 months. In case of switching, the exposure was attributed to the second drug delivery upon the time of switching/swapping. The first drug prescription during the period of the study (1st January 2020 to 31st December 2023) was used as the index date. Prevalent prescriptions before 1st January 2020, but still exposed on that date, were not considered for the purposes of this analysis. Prescriptions were also classified by period (before/after the regulatory agencies’ warnings on 6th July 2021) according to the first prescription of the specific drug. We assessed various time-dependent covariates potentially associated with the use of TNFis or JAKis and considered as a risk factor for CVEs, identified at each new treatment course entry. Age, disease duration (calculated using diagnosis code release dates and classified as 0–2 years, 2–5 years, and >5 years), line of treatment for RA (number of previous different b-DMARDs/JAKis; recall 10 years), previous CVEs (recall 10 years), e-RHD-FI, and hospitalization for any cause (recall 1 year) were assessed. The comorbidities of interest, including hypertension, dyslipidemia, and diabetes mellitus, were identified by analyzing therapeutic prescriptions using the ATC classification system or by considering diagnosis codes or hospitalizations for the aforementioned diseases according to validated algorithms (Generali et al., 2018; Argnani et al., 2021). Glucocorticoids (GCs), non-steroidal anti-inflammatory drugs (NSAIDs), c-DMARDs [including methotrexate (MTX)], and CV drugs (lipid-lowering treatments, platelet aggregation inhibitors, antithrombotic agents, cardiac therapy, anti-hypertensives, anti-diabetic drugs, oral contraceptives, or hormone replacement therapy) were identified as co-medications and defined as any drug delivery 3 months before or after each new treatment course entry, according to the relevant ATC Codes.

### 2.5 Outcomes

We used diagnostic codes (ICD-9-CM) for each CVE and identified them using primary or secondary diagnostic code hospitalizations according to validated algorithms (Tamariz et al., 2012; Cozzolino et al., 2019; Hyeraci et al., 2019; Argnani et al., 2021; Bosco et al., 2021; Kim et al., 2023). The primary outcome was any incident CVE defined as a CVE resulting in hospitalization or emergency department admission. The CVEs included in the present study were sudden cardiac death, myocardial infarction, coronary revascularization, angina, ischemic heart disease, stroke, transient ischemic attack (TIA), congestive heart failure (CHF), peripheral arterial vascular disease (PAVD), deep vein thrombosis (DVT), and pulmonary embolism (PE). Secondary outcomes were subgroups of CVEs, defined as MACEs and TEs. MACEs included sudden cardiac death, non-fatal myocardial infarction or stroke, TIA, and coronary revascularization, as well as CHF or PAVD combined with 30-day event-related mortality (Ytterberg et al., 2022). TEs were defined in the presence of DVT and embolism and/or PE. The diagnostic codes adopted for primary and secondary outcomes are enlisted in Supplementary Table S1.

### 2.6 Statistical analysis

This is a population-based study with a total of 5,563 patients fulfilling the inclusion criteria. Based on the number of TNFi users and JAKi users and the observed rate of CVEs, a non-inferiority test achieves 79% power at a significance level of 0.05 when the hazard ratio (HR) is actually 1 and the non-inferiority margin is 1.8 [pre-specified threshold for non-inferiority in the ORAL Surveillance trial (Ytterberg et al., 2022)], assuming a constant HR and using Cox proportional hazard regression to analyze data.

Baseline characteristics of patients were described by medians and quartiles (first–third quartile, Q1–Q3) and compared using the Kruskal–Wallis rank-sum test for continuous data or by counts and percentages and compared using the chi-square (χ^2^) test for categorical data, according to the first drug exposure during the study period. The observation period started from the index date and ended at the occurrence of the first CVE. Patients were administratively censored at the end of the study (31st December 2023) or when they died or emigrated from the region. Crude incidence rates (IRs) were computed as the total number of events divided by the total time at risk. The 95% confidence intervals (CIs) for the crude incidence rate were calculated from a Poisson distribution. The prescription of non-TNFi/non-JAKi drugs (IL-6i, RTX, and ABT) was also considered in the study but not for comparative purposes as the main interest was on the comparison between JAKis and TNFis.

The risk of CVEs was compared between JAKi and TNFi users using crude and adjusted time-dependent Cox regression analyses with drug exposure described in Section 2.4. Prespecified confounders were age, e-RHD-FI (per 0.1-point increment), gender (male versus female), treatment line (number of previous b/tsDMARD treatment lines equal to 0, 1, 2, or ≥3), and concomitant GCs, NSAIDs, MTX, or CV drugs. Similarly, we assessed the risk of new MACEs and TEs as part of the endpoint analyses, as well as the risk of CVEs among individual JAKi users (JAK1 selective agents upadacitinib and filgotinib, JAK1/2 selective baricitinib, and pan-JAK tofacitinib) compared to that among TNFi users. Furthermore, we tested the interaction between drug treatment and regulatory agency prescription rule changes or patient profiles (age, baseline CV risk, and frailty) on the overall CVE risk. Specifically, the variables assessed for the interaction were the starting date before or after the first pronouncement of the EMA with respect to tofacitinib (06th July 2021), age ≥65 years, e-RHD-FI ≥ 0.056 [defined cut-off of frailty based on the previous report by Rebora et al. (2023)], and the fulfillment of the inclusion criteria of the ORAL Surveillance trial (Ytterberg et al., 2022) or the new PRAC measures (EMA, 2022). The results were presented as HRs and 95% CIs. A sensitivity analysis was performed using a sequential Cox proportional hazard model to estimate the causal effect of drug exposures on CVEs in the presence of time-dependent confounders (Gran et al., 2010). It uses observational data to mimic several randomized controlled trials, in which each trial is based on individuals starting treatment within a certain time interval. Stratified weighted Cox analysis on the joint dataset of all the constructed trials, in which each trial is one stratum, is used for analysis weighted for the inverse probability of censoring. The analyses were performed using SAS software (v9.4) and R (v3.6).

## 3 Results

### 3.1 Descriptive analysis

Patients with the RA diagnosis code, exposed to a TNFi or JAKi course during the period of interest, were included in the analyses. A total of 7,541 therapeutic courses were recorded in 5,563 patients between 1st January 2020 and 31st December 2023 (1,459 days); among them, 2,343 patients were included in the study as TNFi users and 1,443 as JAKi users (Supplementary Figure S1). Table 1 shows the baseline demographic and clinical characteristics of the patients having their first TNFi or JAKi course at study entry, and Supplementary Table S2 shows the characteristics of the patients starting a non-TNFi/non-JAKi drug (1,777 patients). The prevalence of pre-existing CVEs was not significantly different in the JAKi group compared to that in the TNFi group [88 (6.1%) vs. 121 (5.2%); p = 0.251]. Among the single types of pre-existing CVEs, only congestive heart failure (CHF) was significantly more frequent in JAKi users than TNFi users [11 (0.8%) vs. 6 (0.3%); p = 0.044]. The fulfillment of the PRAC measures was mostly present with JAKi therapy [712 (49.3%) vs. 1,070 (45.7%); p = 0.030], while no significant differences emerged for the ORAL Surveillance trial inclusion criteria satisfaction. JAKi users were older than TNFi users [median age 59 years (Q1–Q3 51, 68) and 57 years (Q1–Q3 47, 66); p < 0.001] and shared a longer disease duration and less frequent first-line treatment [775 (53.7%) and 1761 (75.2%); p < 0.001]. Moreover, JAKi users had a higher e-RHD-FI than TNFi users, with e-RHD-FI≥0.056 values in 677 (46.9%) vs. 1,010 (43.1%) (p = 0.024). During the study period, 450 (19.2%) from first TNFi users switched to JAKi users or to other b-DMARD users, while 329 (22.8%) from first JAKi users switched to TNFi users or to other b-DMARD users, with a total exposure of 3,851 person-years (PYs) in TNFi users and 3,173 in JAKi users.

**TABLE 1:** Baseline demographic and clinical characteristics of the patients having their first TNFi or JAKi course at study entry.

Variable of interest	Overall	TNFi	JAKi	p
n	3,786	2,343	1,443	
Male gender, n (%)	886 (23.4)	585 (25.0)	301 (20.9)	0.004
Age, median [Q1, Q3]	58 [49, 67]	57 [47, 66]	59 [51, 68]	<0.001
Age ≥65 years, n (%)	1,146 (30.3)	668 (28.5)	478 (33.1)	0.003
e-RHD-FI, median [Q1, Q3]	0.05 [0.04, 0.08]	0.05 [0.04, 0.06]	0.05 [0.04, 0.08]	0.002
e-RHD-FI ≥ 0.056, n (%)	1,687 (44.6)	1,010 (43.1)	677 (46.9)	0.024
Disease duration, years, n (%)				<0.001
0–2	1,200 (31.7)	830 (35.4)	370 (25.6)	
2–5	635 (16.8)	410 (17.5)	225 (15.6)	
≥5	1951 (51.5)	1,103 (47.1)	848 (58.8)	
Previous treatment lines, n (%)				<0.001
0	2,536 (67.0)	1,761 (75.2)	775 (53.7)	
1	830 (21.9)	434 (18.5)	396 (27.4)	
2	300 (7.9)	105 (4.5)	195 (13.5)	
≥3	120 (3.2)	43 (1.8)	77 (5.3)	
Cardio-cerebrovascular disease, n (%)	209 (5.5)	121 (5.2)	88 (6.1)	0.251
Myocardial infarction, n (%)	18 (0.5)	11 (0.5)	7 (0.5)	1.000
Coronary revascularization, n (%)	32 (0.8)	18 (0.8)	14 (1.0)	0.634
Angina, n (%)	22 (0.6)	13 (0.6)	9 (0.6)	0.960
Ischemic heart disease, n (%)	88 (2.3)	50 (2.1)	38 (2.6)	0.379
Stroke, n (%)	12 (0.3)	9 (0.4)	3 (0.2)	0.523
Transient ischemic attack, n (%)	15 (0.4)	11 (0.5)	4 (0.3)	0.517
Congestive heart failure, n (%)	17 (0.4)	6 (0.3)	11 (0.8)	0.044
Peripheral arterial vascular disease, n (%)	42 (1.1)	21 (0.9)	21 (1.5)	0.151
Deep vein thrombosis and embolism, n (%)	27 (0.7)	15 (0.6)	12 (0.8)	0.631
Pulmonary embolism, n (%)	14 (0.4)	10 (0.4)	4 (0.3)	0.645
Hypertension, n (%)	1,039 (27.4)	608 (25.9)	431 (29.9)	0.010
Diabetes, n (%)	263 (6.9)	157 (6.7)	106 (7.3)	0.489
Dyslipidemia, n (%)	356 (9.4)	209 (8.9)	147 (10.2)	0.215
Previous hospitalizations, n (%)	489 (12.9)	299 (12.8)	190 (13.2)	0.755
Non-steroidal anti-inflammatory drugs, n (%)	1,206 (31.9)	692 (29.5)	514 (35.6)	<0.001
Glucocorticoids, n (%)	1,876 (49.6)	1,100 (46.9)	776 (53.8)	<0.001
c-DMARDs, n (%)	2,261 (59.7)	1,363 (58.2)	898 (62.2)	0.015
Methotrexate, n (%)	1,707 (45.1)	1,043 (44.5)	664 (46.0)	0.386
CV drugs, n (%)	863 (22.8)	473 (20.2)	390 (27.0)	<0.001
ATC, n (%)				-
L04AA29 (tofacitinib)	140 (3.7)	-	140 (9.7)	
L04AA37 (baricitinib)	567 (15.0)	-	567 (39.3)	
L04AA44 (upadacitinib)	376 (9.9)	-	376 (26.1)	
L04AA45 (filgotinib)	360 (9.5)	-	360 (24.9)	
L04AB01 (etanercept)	858 (22.7)	858 (36.6)	-	
L04AB02 (infliximab)	45 (1.2)	45 (1.9)	-	
L04AB04 (adalimumab)	1,070 (28.3)	1,070 (45.7)	-	
L04AB05 (certolizumab pegol)	231 (6.1)	231 (9.9)	-	
L04AB06 (golimumab)	139 (3.7)	139 (5.9)	-	
ORAL criteria, n (%)	1,207 (31.9)	732 (31.2)	475 (32.9)	0.299
PRAC criteria, n (%)	1,782 (47.1)	1,070 (45.7)	712 (49.3)	0.030
Index date after 06th July 2021, n (%)	2,362 (62.4)	1,477 (63.0)	885 (61.3)	0.308

Abbreviations: JAKi, Janus kinase inhibitor; TNFi, tumor necrosis factor inhibitor; e-RHD-FI, electronic-regional healthcare database frailty index; GCs, glucocorticoids; NSAIDs, non-steroidal anti-inflammatory drugs; c-DMARDs, conventional synthetic disease-modifying antirheumatic drugs; CV, cardio-cerebrovascular; ATC, anatomical therapeutic chemical; ORAL, oral rheumatoid arthritis trial; PRAC, Pharmacovigilance Risk Assessment Committee.

### 3.2 Endpoint analyses

A total of 238 new CVEs were recorded, 64 among TNFi and 59 among JAKi users, with 68 new MACEs (24 among TNFi and 18 among JAKi users) and 45 new TEs (9 among TNFi and 13 among JAKi users). The crude IRs for new CVEs were 16.6 (95% CI: 12.8–21.2) and 18.6 (95% CI: 14.2–23.9) per 1,000 PYs among TNFi and JAKi users, respectively. The IRs for new MACEs were 6.2 (95% CI: 3.9–9.3) and 5.7 (95% CI: 3.3–8.9), and the IRs for new TEs were 2.3 (95% CI: 1.1–4.4) and 4.1 (95% CI: 2.2–7.1) among TNFi and JAKi users, respectively (Supplementary Table S3).

**FIGURE 1:**
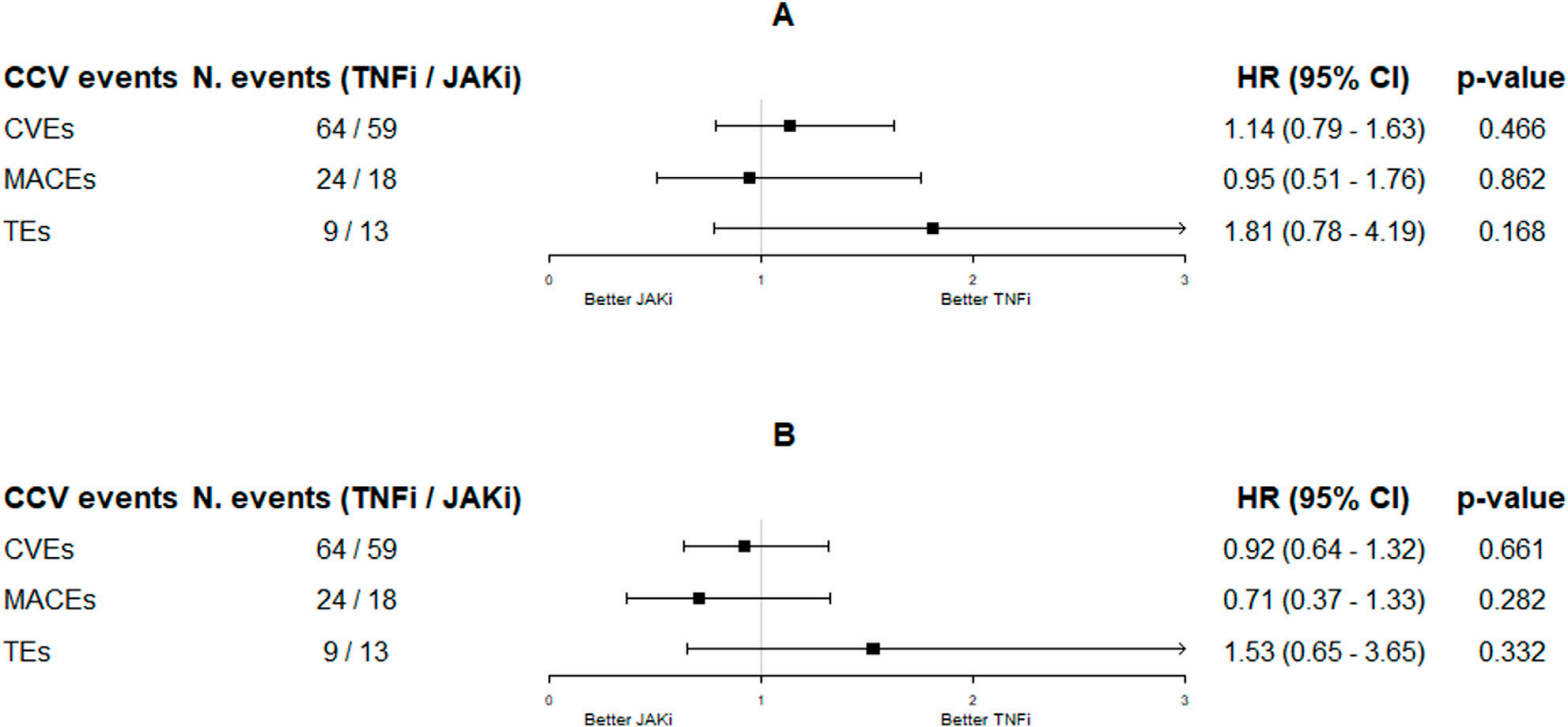
Hazard ratios (95% CI) resulting from the unadjusted [panel **(A)**] and adjusted [panel **(B)**] time-dependent Cox proportional hazard regression models on CCV events by JAKi treatment (TNFi as reference). Adjustments were made by age, e-RHD-FI, gender, treatment line, and assumption of NSAIDs, GCs, MTX, and CV drugs. Abbreviations: CCV, cardio-cerebrovascular; TNFis, tumor necrosis factor inhibitors; JAKis, Janus kinase inhibitors; HR, hazard ratio; CI, confidence interval; CVE, cardio-cerebrovascular event; MACE, major cardiovascular event; TE, thromboembolic event.

The HR for new CVEs was not significantly different in JAKi users compared to that in TNFi users (crude HR: 1.14; 95% CI: 0.79–1.63; adjusted HR: 0.92; 95% CI: 0.64–1.32) (Figure 1). When the sequential Cox proportional hazard regression modeling was performed (Supplementary Table S4), the HR estimate resulted 0.70 (95% CI: 0.33–1.51), with the upper confidence limit still lower than 1.8, for example, the ORAL Surveillance non-inferiority margin (Ytterberg et al., 2022). Similarly, the crude and adjusted HRs for new MACEs were not significantly different in JAKi users compared to those in TNFi users (crude HR: 0.95; 95% CI: 0.51–1.76; adjusted HR: 0.71; 95% CI: 0.37–1.33). The crude HR for new TEs was 1.81 (95% CI: 0.78–4.19) in JAKis vs. TNFis, and the adjusted HR was 1.53 (95% CI: 0.65–3.65) (Figure 1).

In the adjusted analyses (Table 2), each 0.1-point increment in the e-RHD-FI significantly increased the HR for new CVEs (HR: 1.80; 95% CI: 1.48–2.19), MACEs (HR: 1.66; 95% CI: 1.10–2.51), and TEs (HR: 1.69; 95% CI: 1.03–2.78). A similar association was observed for age, male gender, and the second b/tsDMARD treatment line vs. the first one for CVEs and MACEs. Interestingly, MTX co-treatment significantly reduced the risk of MACEs (HR: 0.60; 95% CI: 0.37–0.99), and NSAIDs reduced the risk of CVEs (HR: 0.75; 95% CI: 0.57–0.99) and TEs (HR: 0.36; 95% CI: 0.16–0.80), while GCs significantly increased the risk of TEs (HR: 2.22; 95% CI: 1.14–4.35).

**TABLE 2:** Unadjusted and adjusted time-dependent Cox proportional hazard regression models on CCV events by drug exposure, as reported in Figure 1.

	CCV events											
	CVEs (n = 238)				MACEs (n = 68)				TEs (n = 45)			
	Unadjusted		Adjusted		Unadjusted		Adjusted		Unadjusted		Adjusted	
Parameter	HR (95% CI)	p	HR (95% CI)	p	HR (95% CI)	p	HR (95% CI)	p	HR (95% CI)	p	HR (95% CI)	P
JAKi vs. TNFi	1.14 (0.79–1.63)	0.466	0.92 (0.64–1.32)	0.661	0.95 (0.51–1.76)	0.862	0.71 (0.37–1.33)	0.282	1.81 (0.78–4.19)	0.168	1.53 (0.65–3.65)	0.332
IL-6i/RTX/ABT vs. TNFi	2.26 (1.67–3.07)	<0.001	1.42 (1.04–1.95)	0.027	1.35 (0.78–2.35)	0.286	0.79 (0.46–1.36)	0.394	3.18 (1.47–6.86)	0.003	2.20 (0.97–4.99)	0.059
Age (years)			1.04 (1.02–1.05)	<0.001			1.04 (1.02–1.07)	0.002			1.02 (0.99–1.05)	0.062
eRHD-FI (per 0.1-point increment)			1.80 (1.48–2.19)	<0.001			1.66 (1.10–2.51)	0.016			1.69 (1.03–2.78)	0.037
Sex (M vs. F)			1.63 (1.24–2.15)	<0.001			2.80 (1.74–4.48)	<0.001			0.72 (0.35–1.50)	0.379
Previous treatment lines (1 vs. 0)			1.55 (1.16–2.07)	0.003			2.11 (1.24–3.59)	0.006			1.15 (0.58–2.28)	0.686
Previous treatment lines (2 vs. 0)			1.05 (0.67–1.65)	0.837			1.16 (0.48–2.76)	0.744			1.41 (0.60–3.32)	0.430
Previous treatment lines (≥3 vs. 0)			1.45 (0.90–2.33)	0.126			1.64 (0.70–3.85)	0.254			0.81 (0.22–2.93)	0.749
NSAIDs (Y vs. N)			0.75 (0.57–0.99)	0.048			0.64 (0.38–1.10)	0.107			0.36 (0.16–0.80)	0.012
GC (Y vs. N)			1.20 (0.91–1.57)	0.196			1.15 (0.70–1.87)	0.588			2.22 (1.14–4.35)	0.020
Methotrexate (Y vs. N)			0.86 (0.66–1.12)	0.266			0.60 (0.37–0.99)	0.045			0.75 (0.40–1.39)	0.353
CV drugs (Y vs. N)			2.33 (1.73–3.14)	<0.001			2.59 (1.55–4.34)	<0.001			1.52 (0.75–3.08)	0.250

Abbreviations: CCV, cardio-cerebrovascular; CVE, cardio-cerebrovascular event; MACE, major cardiovascular event; TE, thromboembolic event; HR, hazard ratio; CI, confidence interval; JAKi, Janus kinase inhibitor; TNFi, tumor necrosis factor inhibitor; IL6, interleukin 6; RTX, rituximab; ABT, abatacept; e-RHD-FI, electronic-regional healthcare database frailty index; GCs, glucocorticoids; NSAIDs, non-steroidal anti-inflammatory drugs; CV, cardio-cerebrovascular.

### 3.3 Secondary analyses

#### 3.3.1 Specific JAKi drugs

**FIGURE 2:**
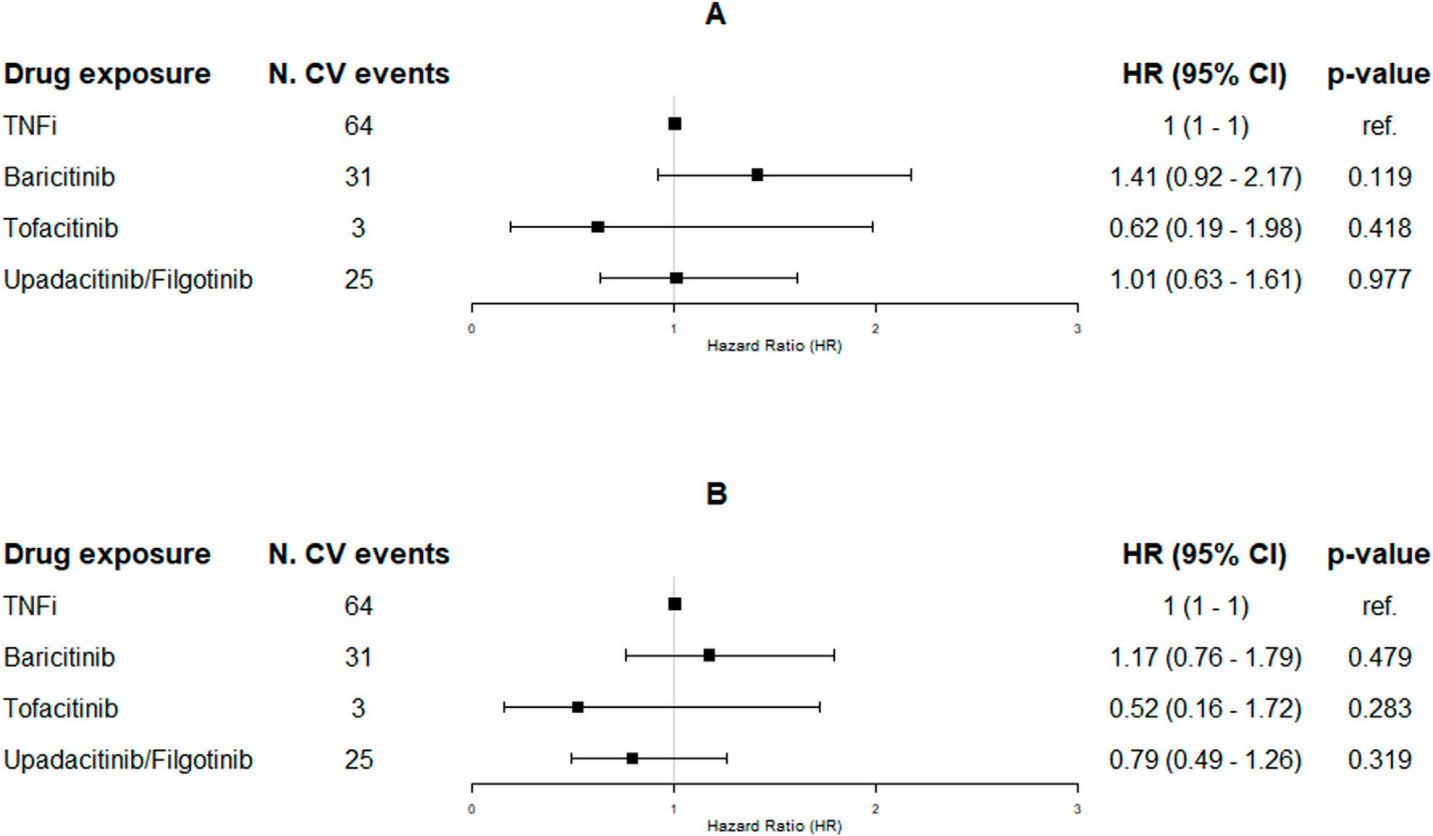
Hazard ratios (95% CI) resulting from the unadjusted [panel **(A)**] and adjusted [panel **(B)**] time-dependent Cox proportional hazard regression models on CV events by the subgroups of JAKi exposure (TNFi as reference). Adjustments were made by age, e-RHD-FI, gender, treatment line, and assumption of NSDAIDs, GCs, MTX, and CV drugs. Abbreviations: CV, cardio-cerebrovascular; TNFis, tumor necrosis factor inhibitors; JAKi, Janus kinase inhibitors; HR, hazard ratio; CI, confidence interval.

Among JAKi users, 140 (9.7%) entered the study using tofacitinib, 567 (39.3%) using baricitinib, 360 (24.9%) using upadacitinib, and 376 (26.1%) using filgotinib (Table 1). The crude IRs for new CVEs were 22.7 (95% CI: 15.4–32.1), 9.9 (95% CI: 2.1–28.9), and 16.7 (95% CI: 10.8–24.6) per 1,000 PYs among baricitinib, tofacitinib, and upadacitinib/filgotinib users, respectively (Supplementary Table S5). Figure 2 shows the association between the specific JAKis and the occurrence of new CVEs during the follow-up period. No single JAKi was significantly associated with the risk of new CVEs in crude and adjusted analyses compared to TNFi users. Again, it was the patients’ profile that was mostly associated with the outcome of interest (specifically age, male gender, e-RHD-FI, and treatment line; Supplementary Table S6).

#### 3.3.2 Interaction between the regulatory agencies’ warnings and patients’ profiles on the risk of new CVEs

**FIGURE 3:**
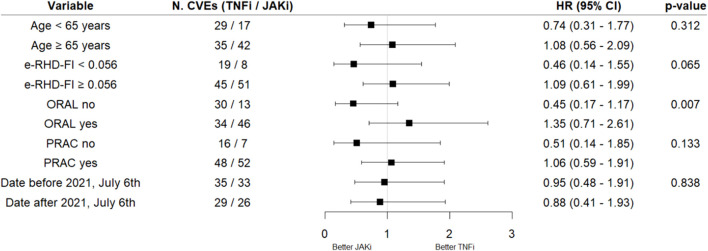
Forest plot of hazard ratios (95% CI) resulting from pairwise comparisons based on adjusted time-dependent Cox proportional hazard regression models on CV events by the interaction between JAKi exposure and variables of interest (TNFi as the reference). Adjustments were made by age, e-RHD-FI, gender, treatment line, and assumption of NSDAIDs, GCs, MTX, and CV drugs. P-values were derived from the interaction between JAKi exposure and variables of interest. Abbreviations: CVE, cardio-cerebrovascular event; TNFis, tumor necrosis factor inhibitors; JAKis, Janus kinase inhibitors; HR, hazard ratio; CI, confidence interval; e-RHD-FI, electronic-regional healthcare database frailty index; ORAL, oral rheumatoid arthritis trial; PRAC, Pharmacovigilance Risk Assessment Committee.

Out of 1,443 JAKi courses, 558 (38.7%) started before 06th July 2021, while 885 (61.3%) started after this date (Table 3). The number of tofacitinib treatment courses reduced in the second part of the study period [117 (21.0%) vs. 23 (2.6%)]. Treatment courses with JAKis before EMA pronouncement were administered to patients with a longer disease duration (p = 0.004), mostly in combination with GCs [334 (59.9%) vs. 442 (49.9%), p < 0.001] or NSAIDs [384 (68.8%) vs. 514 (58.1%); p < 0.001]. Among all the CV risk factors assessed, only hypertension was significantly more common before 06th July 2021 [189 (33.9%) vs. 242 (27.3%); p = 0.010]. The distribution of previous CVEs did not vary between the groups, despite a non-significant increase in the number of previous venous thromboembolisms (VTEs) after 06th July 2021 [11 (1.2%) vs. 1 (0.2%); p = 0.062]. When assessing the interaction between the period (before or after 06th July 2021) and the treatment with JAKi on the risk of new CVEs, no significant interaction was observed (p = 0.838) (Figure 3).

**TABLE 3:** Frequency of RA patients treated with JAKis by the index date.

Variable of interest	Overall	Before 06th July 2021	After 06th July 2021	p
n	1,443	558	885	
Male gender, n (%)	301 (20.9)	122 (21.9)	179 (20.2)	0.497
Age, median [Q1, Q3]	59 [51, 68]	59 [51, 68]	59 [51, 67]	0.847
Age ≥65 years, n (%)	478 (33.1)	201 (36.0)	277 (31.3)	0.072
e-RHD-FI, median [Q1, Q3]	0.05 [0.04, 0.08]	0.05 [0.04, 0.08]	0.05 [0.04, 0.08]	0.050
e-RHD-FI ≥ 0.056, n (%)	677 (46.9)	266 (47.7)	411 (46.4)	0.688
Disease duration (years), n (%)				0.004
0–2	370 (25.6)	170 (30.5)	200 (22.6)	
2–5	225 (15.6)	83 (14.9)	142 (16.0)	
≥5	848 (58.8)	305 (54.7)	543 (61.4)	
Previous treatment lines, n (%)				0.793
0	775 (53.7)	303 (54.3)	472 (53.3)	
1	396 (27.4)	147 (26.3)	249 (28.1)	
2	195 (13.5)	75 (13.4)	120 (13.6)	
≥3	77 (5.3)	33 (5.9)	44 (5.0)	
Cardio-cerebrovascular disease, n (%)	88 (6.1)	38 (6.8)	50 (5.6)	0.433
Myocardial infarction, n (%)	7 (0.5)	2 (0.4)	5 (0.6)	0.872
Coronary revascularization, n (%)	14 (1.0)	5 (0.9)	9 (1.0)	1.000
Angina, n (%)	9 (0.6)	5 (0.9)	4 (0.5)	0.484
Ischemic heart disease, n (%)	38 (2.6)	19 (3.4)	19 (2.1)	0.199
Stroke, n (%)	3 (0.2)	1 (0.2)	2 (0.2)	1.000
Transient ischemic attack, n (%)	4 (0.3)	3 (0.5)	1 (0.1)	0.327
Congestive heart failure, n (%)	11 (0.8)	7 (1.3)	4 (0.5)	0.163
Peripheral arterial vascular disease, n (%)	21 (1.5)	11 (2.0)	10 (1.1)	0.283
Deep vein thrombosis, n (%)	12 (0.8)	1 (0.2)	11 (1.2)	0.062
Pulmonary embolism, n (%)	4 (0.3)	2 (0.4)	2 (0.2)	1.000
Hypertension, n (%)	431 (29.9)	189 (33.9)	242 (27.3)	0.010
Diabetes, n (%)	106 (7.3)	49 (8.8)	57 (6.4)	0.120
Dyslipidemia, n (%)	147 (10.2)	59 (10.6)	88 (9.9)	0.767
Previous hospitalization, n (%)	190 (13.2)	73 (13.1)	117 (13.2)	1.000
Non-steroidal anti-inflammatory drugs, n (%)	514 (35.6)	215 (38.5)	299 (33.8)	0.076
Glucocorticoids, n (%)	776 (53.8)	334 (59.9)	442 (49.9)	<0.001
c-DMARDs, n (%)	898 (62.2)	384 (68.8)	514 (58.1)	<0.001
Methotrexate, n (%)	664 (46.0)	275 (49.3)	389 (44.0)	0.054
CV drugs, n (%)	390 (27.0)	166 (29.7)	224 (25.3)	0.074
ATC, n (%)				<0.001
L04AA29 (tofacitinib)	140 (9.7)	117 (21.0)	23 (2.6)	
L04AA37 (baricitinib)	567 (39.3)	343 (61.5)	224 (25.3)	
L04AA44 (upadacitinib)	376 (26.1)	81 (14.5)	295 (33.3)	
L04AA45 (filgotinib)	360 (24.9)	17 (3.0)	343 (38.8)	
ORAL criteria, n (%)	475 (32.9)	197 (35.3)	278 (31.4)	0.140
PRAC criteria, n (%)	712 (49.3)	289 (51.8)	423 (47.8)	0.154

Abbreviations: JAKi, Janus kinase inhibitor; e-RHD-FI, electronic-regional healthcare database frailty index; GCs, glucocorticoids; NSAIDs, non-steroidal anti-inflammatory drugs; c-DMARDs, conventional synthetic disease-modifying antirheumatic drugs; CV, cardio-cerebrovascular; ATC, anatomical therapeutic chemical; ORAL, oral rheumatoid arthritis trial; PRAC, Pharmacovigilance Risk Assessment Committee.

**FIGURE 4:**
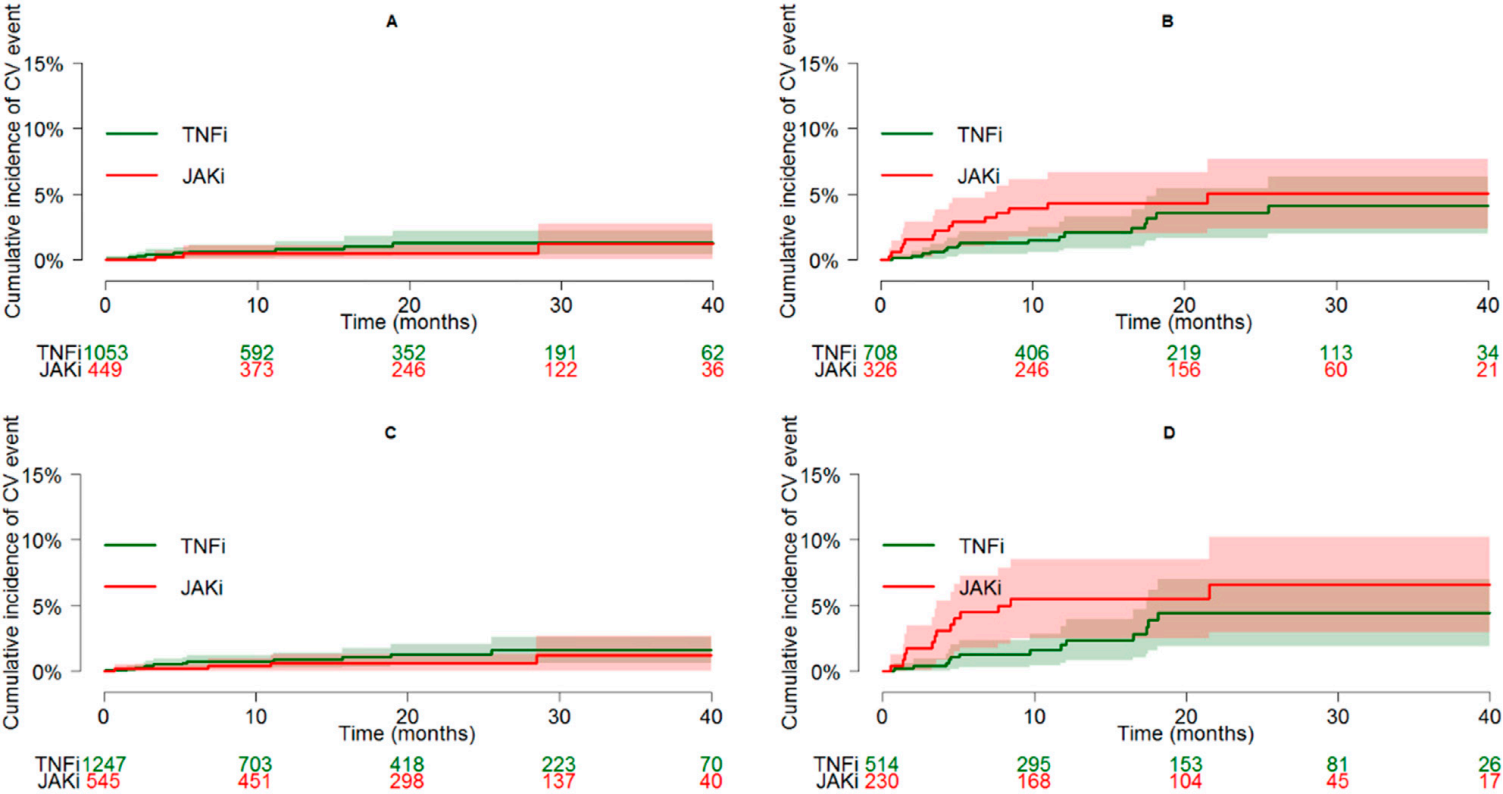
Cumulative incidence plots (and 95% CI bands) of CVE in patients on first-line TNFi or JAKi, according to specific characteristics: e-RHD-FI < 0.056 [panel **(A)**], e-RHD-FI ≥ 0.056 [panel **(B)**], not fulfilling ORAL inclusion criteria [panel **(C)**], and fulfilling ORAL inclusion criteria [Panel **(D)**]. Abbreviations: CV, cardio-cerebrovascular; TNFis, tumor necrosis factor inhibitors; JAKis, Janus kinase inhibitors; CI, confidence interval; e-RHD-FI, electronic-regional healthcare database frailty index.

Separate estimates of the HR of JAKi exposure versus TNFis in different subgroups are reported in Figure 3. The HR was higher in frail patients (e-RHD-FI≥0.056), although the interaction on the risk of new CVEs was not statistically significant (p = 0.065). Similarly, no significant interaction was observed between age and JAKis on the risk of new CVEs (p = 0.312). After stratifying the therapeutic courses based on the fulfillment of the inclusion criteria of the ORAL Surveillance trial or to the new PRAC suggestions, a significant interaction was found for ORAL but not for PRAC (p = 0.007 and 0.133, respectively). In particular, the HR in patients fulfilling the ORAL inclusion criteria was 1.35 (0.71–2.61), crossing the non-inferiority margin. The cumulative incidences of CVEs, restricted to the first-line TNFi or JAKi, and stratified by e-RHD-FI ≥ 0.056 or the fulfillment of ORAL surveillance inclusion criteria are reported in Figure 4.

## 4 Discussion

The present study aimed to investigate the association of JAKis, in comparison with TNFis, with the risk of CVEs in a large population of individuals with RA. Overall, our results indicate that the exposure to JAKis did not exhibit a significantly increased risk of CVEs compared to TNFis. Similarly, the risk of MACEs was not significantly different between the groups, while TEs occurred more frequently in JAKi-treated patients, although the difference was not significant. Notably, our data suggest that frailty, as assessed by a validated electronic frailty index (e-RHD-FI), and baseline CV risk, as the fulfillment of the ORAL surveillance inclusion criteria, identify subgroups of patients with a potentially higher risk of CVEs in JAKi-exposed patients.

Our primary findings were consistent with the results of several real-world studies. In the STAR-RA study, conducted in 102,263 patients with RA treated in a real-world setting, tofacitinib was not associated with an increased risk of CVEs when compared to TNFis (pooled weighted HR: 1.01; 95% CI: 0.83–1.23) (Khosrow-Khavar et al., 2022). Similarly, in several AHD-based studies, no significant risk of CVEs was observed with tofacitinib and other JAKis (mostly baricitinib and upadacitinib) compared to c-DMARDs (Ozen et al., 2021; Ahn et al., 2023; Frisell et al., 2023; Popa et al., 2023; Ma et al., 2024; Tong et al., 2023). Considering the risk of MACEs, the ORAL Surveillance trial indicated an increased risk of events with tofacitinib versus TNFis, albeit not statistically significant (tofacitinib combined dose HR: 1.33; 95% CI: 0.91–1.94) (Ytterberg et al., 2022). This trial, specifically, did not show non-inferiority of tofacitinib since the upper boundary of the two-sided 95% CI was higher than the pre-specified threshold of 1.8. In our results, instead, the upper limit of the 95% CI was less than 1.8 (adjusted HR for MACEs: 0.71; 95% CI: 0.37–1.33), indicating the non-inferiority of JAKis. Considering real-life experiences, our results are similar to those of a population-based cohort study using data from the French national health data system, which included 15,835 patients, among which 8,481 were exposed to JAKis and 7,354 to adalimumab: the risk of MACEs did not significantly differ between those initiating a JAKi or adalimumab (adjusted HR: 1.0; 95% CI: 0.7–1.5) (Hoisnard et al., 2023), even when considering patients with at least one CV risk factor who were >50 years. In the ARTIS, CORRONA, and RABBIT registries, no clear difference in the rate of MACEs was observed in baricitinib or tofacitinib versus b-DMARDs users (Kremer et al., 2021; Frisell et al., 2023; Meissner et al., 2023; Mok et al., 2023). Recent systematic literature reviews and meta-analyses confirmed these reassuring data both in RCTs and observational studies (Wei et al., 2023; Partalidou et al., 2024), despite suggesting a higher mortality rate for all causes in the case of tofacitinib use in RCTs (Wei et al., 2023).

The assessment of the risk of TEs deserves attention. In our data, the crude incidence rate of TEs was higher among patients exposed to a JAKi, and the adjusted HR for new TEs was higher in ts-DMARDs- vs. TNFi users (HR = 1.53; 95% CI = 0.65–3.65). Albeit not primarily powered for this analysis and considering the relatively low incidence of TEs in our sample (reducing the *post hoc* power to 35%), our results indicate that the non-inferiority cut off of 1.8 was intercepted. These data suggest a confirmation for the increase in the incidence of VTE, DVT, and PE with tofacitinib observed in the ORAL Surveillance study (Ytterberg et al., 2022; Charles-Schoeman et al., 2023; Kristensen et al., 2023) and with baricitinib in *post hoc* analyses and real-life studies (Taylor et al., 2019; Salinas et al., 2023). We have to underline that AHD-based studies are not able to intercept clinical variables like disease activity, which could have a role in VTEs occurrence (Charles-Schoeman et al., 2024), but we confirmed the role of GCs in worsening CV outcomes of RA subjects (Coburn et al., 2024). On the other hand, we performed a sensitivity analysis adjusting for the presence of previous DVTs, and the HR did not vary (HR: 1.53; 95% CI: 0.64–3.63). So far, several post-marketing studies have evaluated the association between VTE risks in patients treated with JAKis (Desai et al., 2019; 2022; Setyawan et al., 2021; Gouverneur et al., 2022; Hoisnard et al., 2023; Min et al., 2023). Although a recent meta-analysis did not highlight an increased risk of TEs with JAKis vs. TNFis (Partalidou et al., 2024), some exceptions should be underlined, as in a nationwide study from Sweden, in which the risk of VTE in patients with RA treated with baricitinib and tofacitinib was significantly higher than the risk in those treated with b-DMARDs (adjusted HR with JAKis versus TNFis = 1.73; 95% CI = 1.24–2.42 for VTE, HR = 3.21 and 95% CI = 2.11–4.88 for PE, and HR = 0.83; 95% CI = 0.47–0.45 for DVT) (Molander et al., 2023).

Real-world data regarding the comparative CV safety of individual JAKis are progressively increasing. To the best of our knowledge, only two AHD-based studies focused on CV outcomes with all the four JAKis approved by the EMA for the systemic management of RA (Bower et al., 2023; Sakai et al., 2024). In the first study, similarly to our results, none of the single JAKis demonstrated a significant association with CVE occurrence, although we found a numerically lower HR for tofacitinib than that for upadacitinib/filgotinib and baricitinib (Bower et al., 2023). In the study by Sakai et al., instead, no formal comparison was performed among individual drugs (Sakai et al., 2024). Given the study period taken in examination for our analysis, it is expected that new data may upgrade the information for the most recently released JAKis (upadacitinib and filgotinib) (Benucci et al., 2023; Favalli et al., 2023; Hayashi et al., 2023; Lanzillotta et al., 2023; Meissner et al., 2023; Yoshida et al., 2023). In fact, a recent analysis of several European registries suggested different behaviors of single JAKis, with TNFis having a lower risk of drug discontinuation for adverse events (AEs) than tofacitinib but higher than those of baricitinib and other JAKis (Aymon et al., 2023). However, since the rules driving JAKi prescription have been modified applying EMA pronouncement, a possible significantly different treatment attitude toward JAKis prescription should be considered for the interpretation of future studies.

Information partially lacking in AHD-based studies in RA refers to the possible consequences that the first pronouncement of the EMA regarding the safety profile of tofacitinib had on the CV safety of JAKis, reflecting changed prescription attitudes. We performed a secondary analysis by stratifying treatment courses started before and after 06th July 2021. As expected, the number of prescribed tofacitinib courses dramatically decreased. This could be a consequence of the safety warning, but also of a reduction in prescription rates following new JAKi release for the same indication. Here, JAKi courses started after 2021 were administered to patients with longer disease duration, although a significant difference did not emerge with respect to age and other CV risk factors. Patients’ characteristics slightly changed before and after this regulatory milestone. It can be speculated that the earlier period included a heterogeneous group of patients, comprising those on subsequent treatment lines, often older and with more comorbidities, as well as younger patients starting their first line of targeted DMARD therapy, likely influenced by the administration route of JAKis. In contrast, during the later period, JAKis were often initiated in patients with limited therapeutic alternatives, leading to an enrichment of individuals with more complex medical profiles and concomitant conditions and only partially with a lower CV risk (Strunz et al., 2024). This hypothesis may explain the comparable proportion of patients with high baseline CV risk in the JAKi group compared with the TNFi group across the two periods, as well as the relatively modest decrease in the baseline CV risk observed in JAKi users after July 2021. In addition, the period did not appear to modify the association between JAKis and CVEs, in line with a Dutch AHD-based study considering tofacitinib and baricitinib (Popa et al., 2023). The possibility to further stratify treatment courses started before and after 23rd January 2023, the date of the second EMA warning referred to all the JAKis approved for RA, should be assessed in future studies to evaluate the weight of possible selection bias.

Even the other characteristics assessed [age >65 years or the measures suggested by the PRAC of EMA (EMA, 2022)] did not modify the association between JAKis and new CVE occurrence, while the satisfaction of the Oral Surveillance trial inclusion criteria (Ytterberg et al., 2022) appeared to modify it, with a higher HR for JAKis, as compared with TNFi, in ORAL-like patients (although the 95% CI of this HR included the unit), with a relative increase in the risk comparable with the ORAL Surveillance trial. This reinforces the hypothesis that it is the patient’s profile rather than the specific drug that impacts on the risk (Argnani et al., 2021; Ahn et al., 2023; Kristensen et al., 2023). Moreover, here, we adopted the e-RHD-FI, a 40-item frailty index developed using electronic-regional health databases (e-RHDs), which showed a good performance in predicting in-hospital and 30-day mortalities, risk of hospital admission, and worsening on the WHO clinical scale in COVID-19 patients (Rebora et al., 2023). The adoption of a different claim-based FI that estimates deficit-accumulation using claim data was also performed in a recent AHD-based study, assessing the risk of infections in RA patients receiving JAKis or TNFis (Singh et al., 2024). However, to the best of our knowledge, this is the first study assessing the influence of frailty on the CV risk of JAKis in AHD-based studies. Again, in our work, it was the value of the e-RHD-FI itself that was associated with CVE occurrence, as well as with MACEs and TEs, suggesting that frailty contributed to an increased risk irrespective of the treatment delivery. In addition, this was confirmed by interaction analyses, which showed that the interaction between the eRHD-FI and JAKi treatment on the risk of CVEs was not significant. Furthermore, it is interesting to note that the HR estimates in frail patients were much higher than in the other patients, and the confidence interval exceeded 1.8 (Figure 3). Consequently, the assessment of frailty should become increasingly common in rheumatology to estimate the risk of AEs, given its multidimensional nature, which serves as a proxy for biological age (Howlett et al., 2021; Salaffi et al., 2023).

Limitations of this study include its retrospective nature and, intrinsic in the study design, the absence of clinical outcomes (e.g., disease activity or radiological data), the lack of control of data collected for non-clinical purposes, as well as the lack of information regarding the smoking status of the subjects enrolled, which formally prevented the possibility to completely overlap with the inclusion criteria of the Oral Surveillance trial (Ytterberg et al., 2022) and with the measures suggested by the PRAC of the EMA (EMA, 2022). However, this is in line with other AHD-based studies assessing the CV risk of DMARDs (Setyawan et al., 2021; Desai et al., 2022). We adjusted for prespecified confounders, but confounding of unmeasured factors (e.g., inflammatory markers, smoking status, and obesity, which could have an obvious role on CV risk determination) could not be excluded, and similarly, we were not able to collect different outcomes, like carotid plaque progression or modification in validated CV risk scores; these are common limitations of observational studies, particularly in the case of AHD-based studies which formally lack a number of clinical variables (Johnson and Nelson, 2013). However, we decided to focus on the same outcomes of the Oral Surveillance trial (Ytterberg et al., 2022), and we did not expect a significant different distribution of such CV risk factors between the populations, at least before 06th July 2021. Moreover, AHD-based studies are powerful instruments to evaluate the implications of clinical decisions on a large scale, and they are increasingly adopted to evaluate patient profiles, including frailty (Kim et al., 2018; Orkaby et al., 2019; Rebora et al., 2023; Singh et al., 2024). Again, we adjusted for concomitant GC treatment, but we lack information on the GC dosage. Finally, since AHDs reflect drug delivery rather the specialists’ “prescription” habits or the real patients’ adherence (Silvagni et al., 2018), this could result in a difference between the rates of prescribed therapies and the rates of drug usage. Anyway, AHDs are commonly used as good instruments to estimate drug prescriptions and exposures (Cadarette and Wong, 2015), and our data align with similar reports (Bower et al., 2023; Sakai et al., 2024). Conversely, relevant strengths of our study include the large sample size, the adoption of an FI specifically validated for the use in AHDs (Rebora et al., 2023), the possibility to assess treatment courses pertaining to all the currently EMA-approved JAKis, considering incident patients with time-dependent covariates, and the stratification for courses started before and after the first EMA pronouncement regarding tofacitinib safety.

In conclusion, this AHD-based study highlighted no significantly increased risk of CVEs or MACEs for JAKis with respect to TNFis, with some red flags confirmed for TEs, and no single JAKi emerged over the others in increasing this risk. The CV risk remains mainly driven by the patient profiles. The frailty, in parallel with baseline CV risk, emerged as an important determinant of CVEs, MACEs, and TEs. These aspects should be considered when clinically assessing RA patients on a ts/b-DMARD therapy, as well as in the process of randomized controlled trial design.

## Data Availability

The data analyzed in this study was obtained from Regione Lombardia, the following restrictions apply: proper agreement with Regione Lombardia for a specific project. Requests to access these datasets should be directed to the DG Welfare of Regione Lombardia.
