# Supplementary material for: Frailty and cardiovascular safety of JAK inhibitors versus TNF inhibitors in rheumatoid arthritis: a real-world comparative study of drug effects and patient profiles
Source: Front Pharmacol. 2025 Apr 25;16:1565909. doi: 10.3389/fphar.2025.1565909 (PMC12062075; doi:10.3389/fphar.2025.1565909)
Supplement: Supplementary file 2 [file DataSheet1.pdf]

Supplementary Materials

Figure S1. Flow chart of patients' selection.

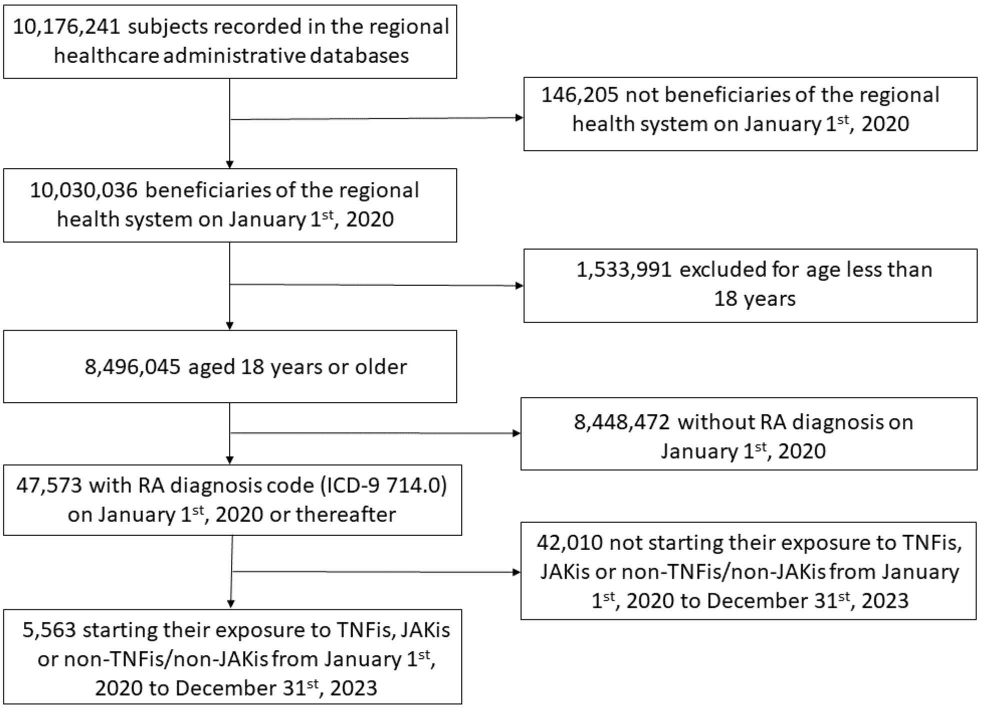

Table S1. List of CVEs and related codes.

	Hospital Discharge Form (HDF)			Emergency Department (ED)
Cardio-cerebrovascular events (CVEs)	***ICD9-CM DX***	***ICD9-CM Proc.***	***DRG-24 code***	***ICD9-CM DX***
Sudden cardiac death + death during hospitalization	*427.5**			*427.5**
Myocardial Infarction (fatal and non-fatal)	*410**		*121, 122, 123, 140*	*410**
All Coronary Revascularization		*36**		
Unstable Angina	*411*, 413**			*411*, 413**
New Ischemic Heart Disease	*414**			*414**
Stroke (fatal and non-fatal)	*431*, 434**			*431*, 434**
Transient Ischemic Attack (TIA)	*435**			*435**
Congestive Heart Failure (CHF)	*428**		*127*	*428**
Peripheral Arterial Vascular Disease (PAVD)	*441*, 444**	*38**		*441*, 444**
Deep Vein Thrombosis and embolism	*451*, 453**			*451*, 453**
Pulmonary Embolism	*415**			*415**
Major Cardiovascular Events (MACEs)				
Sudden cardiac death + death during hospitalization	*427.5**			*427.5**
Myocardial Infarction (non-fatal)	*410**		*121, 122, 123, 140*	*410**
All Coronary Revascularization + death within 30 days		*36**		
Stroke (non-fatal)	*431*, 434**			*431*, 434**
Transient Ischemic Attack (TIA)	*435**			*435**
Congestive Heart Failure (CHF) + death within 30 days	*428**		*127*	*428**
Peripheral Arterial Vascular Disease (PAVD) + death within 30 days	*441*, 444**	*38**		*441*, 444**
Thromboembolic events (TEs)				
Deep Vein Thrombosis and embolism	*451*, 453**			*451*, 453**
Pulmonary Embolism	*415**			*415**

Table S2. Baseline demographic and clinical characteristics of the patients having their 1st TNFi, JAKi or IL-6i / RTX / ABT course at study entry.

Variable of interest	Overall	IL-6i / RTX / ABT	TNFi	JAKi
n	5563	1777	2343	1443
Male gender, n (%)	1349 (24.2)	463 (26.1)	585 (25.0)	301 (20.9)
Age, median [Q1, Q3]	59 [51, 69]	64 [54, 72]	57 [47, 66]	59 [51, 68]
Age ≥ 65 years, n (%)	1997 (35.9)	851 (47.9)	668 (28.5)	478 (33.1)
e-RHD-FI, median [Q1, Q3]	0.05 [0.04, 0.08]	0.06 [0.04, 0.09]	0.05 [0.04, 0.06]	0.05 [0.04, 0.08]
e-RHD-FI ≥ 0.056, n (%)	2762 (49.6)	1075 (60.5)	1010 (43.1)	677 (46.9)
Disease duration (years), n (%)				
0 – 2	1671 (30.0)	471 (26.5)	830 (35.4)	370 (25.6)
2 – 5	883 (15.9)	248 (14.0)	410 (17.5)	225 (15.6)
≥ 5	3009 (54.1)	1058 (59.5)	1103 (47.1)	848 (58.8)
Previous treatment lines, n (%)				
0	3678 (66.1)	1142 (64.3)	1761 (75.2)	775 (53.7)
1	1253 (22.5)	423 (23.8)	434 (18.5)	396 (27.4)
2	439 (7.9)	139 (7.8)	105 (4.5)	195 (13.5)
≥ 3	193 (3.5)	73 (4.1)	43 (1.8)	77 (5.3)
Cardio-cerebrovascular disease, n (%)	433 (7.8)	224 (12.6)	121 (5.2)	88 (6.1)
Myocardial infarction, n (%)	42 (0.8)	24 (1.4)	11 (0.5)	7 (0.5)
Coronary revascularization, n (%)	66 (1.2)	34 (1.9)	18 (0.8)	14 (1.0)
Angina, n (%)	41 (0.7)	19 (1.1)	13 (0.6)	9 (0.6)
Ischemic heart disease, n (%)	159 (2.9)	71 (4.0)	50 (2.1)	38 (2.6)
Stroke, n (%)	34 (0.6)	22 (1.2)	9 (0.4)	3 (0.2)
Transient ischemic attack, n (%)	29 (0.5)	14 (0.8)	11 (0.5)	4 (0.3)
Congestive heart failure, n (%)	56 (1.0)	39 (2.2)	6 (0.3)	11 (0.8)
Peripheral arterial vascular disease, n (%)	98 (1.8)	56 (3.2)	21 (0.9)	21 (1.5)
Deep vein thrombosis and embolism, n (%)	48 (0.9)	21 (1.2)	15 (0.6)	12 (0.8)
Pulmonary embolism, n (%)	35 (0.6)	21 (1.2)	10 (0.4)	4 (0.3)
Hypertension, n (%)	1678 (30.2)	639 (36.0)	608 (25.9)	431 (29.9)
Diabetes, n (%)	439 (7.9)	176 (9.9)	157 (6.7)	106 (7.3)
Dyslipidaemia, n (%)	595 (10.7)	239 (13.4)	209 (8.9)	147 (10.2)
Previous hospitalizations, n (%)	855 (15.4)	366 (20.6)	299 (12.8)	190 (13.2)
Non-steroidal anti-inflammatory drugs, n (%)	1729 (31.1)	523 (29.4)	692 (29.5)	514 (35.6)
Glucocorticoids, n (%)	2803 (50.4)	927 (52.2)	1100 (46.9)	776 (53.8)
c-DMARDs, n (%)	3181 (57.2)	920 (51.8)	1363 (58.2)	898 (62.2)
Methotrexate, n (%)	2362 (42.5)	655 (36.9)	1043 (44.5)	664 (46.0)
CV drugs, n (%)	1417 (25.5)	554 (31.2)	473 (20.2)	390 (27.0)
ATC, n (%)				
L01FA01 (rituximab)	338 (6.1)	338 (19.0)	-	-
L04AA24 (abatacept)	781 (14.0)	781 (44.0)	-	-
L04AA29 (tofacitinib)	140 (2.5)	-	-	140 (9.7)
L04AA37 (baricitinib)	567 (10.2)	-	-	567 (39.3)
L04AA44 (upadacitinib)	376 (6.8)	-	-	376 (26.1)
L04AA45 (filgotinib)	360 (6.5)	-	-	360 (24.9)
L04AB01 (etanercept)	858 (15.4)	-	858 (36.6)	-

L04AB02 (Infliximab)	45 (0.8)	-	45 (1.9)	-
L04AB04 (adalimumab)	1070 (19.2)	-	1070 (45.7)	-
L04AB05 (certolizumab pegol)	231 (4.2)	-	231 (9.9)	-
L04AB06 (golimumab)	139 (2.5)	-	139 (5.9)	-
L04AC07 (tocilizumab)	460 (8.3)	460 (25.9)	-	-
L04AC14 (sarilumab)	198 (3.6)	198 (11.1)	-	-
ORAL criteria = n (%)	1989 (35.8)	782 (44.0)	732 (31.2)	475 (32.9)
PRAC criteria = n (%)	2912 (52.3)	1130 (63.6)	1070 (45.7)	712 (49.3)
Index date after July 06th, 2021, n (%)	3535 (63.5)	1173 (66.0)	1477 (63.0)	885 (61.3)

Abbreviations: *IL6, Interleukin 6; RTX, Rituximab; ABT, Abatacept; JAKi, Janus Kinase inhibitors; TNFi, Tumor Necrosis Factor inhibitors; e-RHD-FI, electronic-regional healthcare database frailty index; GCs, glucocorticoids; NSAIDs, not steroidal anti-inflammatory drugs; c-DMARDs, conventional synthetic disease-modifying antirheumatic drugs; CV, cardio-cerebrovascular; ATC, Anatomical Therapeutic Chemical; ORAL, Oral Rheumatoid Arthritis Trial; PRAC, Pharmacovigilance Risk Assessment Committee.*

Table S3. Crude incidence rates of CVEs in the TNFis and JAKis users (per year, per 1,000 person).

**Drug exposure**	**TNFis (person-years = 3,851)**		**JAKis (person-years = 3,173)**	
**Events**	**N. events**	**IR (95% CI)**	**N. events**	**IR (95% CI)**
CVEs	64	16.6 (12.8 – 21.2)	59	18.6 (14.2 – 23.9)
MACEs	24	6.2 (3.9 – 9.3)	18	5.7 (3.3 – 8.9)
TEs	9	2.3 (1.1 – 4.4)	13	4.1 (2.2 – 7.1)

List of abbreviations: *CVEs, cardio-cerebrovascular events; TNFis, Tumor Necrosis Factor inhibitors; JAKis, Janus Kinase inhibitors; IR, incidence rate; CI, confidence interval; MACEs, major cardiovascular events; TEs, thromboembolic events.*

Table S4. Adjusted sequential Cox proportional hazard regression models on CVEs by drug exposure.

Parameter	HR (95%CI)	p
JAKi vs TNFi	0.70 (0.33 – 1.51)	0.368
IL-6i / RTX / ABT vs TNFi	1.39 (0.83 – 2.32)	0.207
Age (years)	1.05 (1.03 – 1.07)	<0.001
eRHD-FI (per 0.1-point increment)	2.36 (1.77 – 3.14)	<0.001
Sex (M vs F)	1.84 (1.17 – 2.90)	0.008
Previous treatment lines (1 vs 0)	3.06 (1.87 – 4.99)	<0.001
Previous treatment lines (2 vs 0)	1.36 (0.66 – 2.79)	0.402
Previous treatment lines (≥3 vs 0)	2.01 (0.99 – 4.06)	0.053
NSAIDs (Y vs N)	1.08 (0.68 – 1.71)	0.747
GC (Y vs N)	1.86 (1.16 – 2.98)	0.010
Methotrexate (Y vs N)	0.77 (0.50 – 1.19)	0.242
CV drugs (Y vs N)	2.11 (1.39 – 3.19)	<0.001

Abbreviations: *CVE, cardio-cerebrovascular event; HR, hazard ratio; CI, confidence interval; JAKi, Janus Kinase inhibitors; TNFi, Tumor Necrosis Factor inhibitors; IL6, Interleukin 6; RTX, Rituximab; ABT, Abatacept; e-RHD-FI, electronic-regional healthcare database frailty index; GCs, glucocorticoids; NSAIDs, not steroidal anti-inflammatory drugs; CV, cardio-cerebrovascular.*

Table S5. Crude incidence rates of CVEs in the subgroups of JAKis users (per year, per 1,000 person).

**JAKis exposure**	**Baricitinib (person-years = 1,369)**		**Tofacitinib (person-years = 303)**		**Upadacitinib/Filgotinib (person-years = 1,501)**	
**Events**	**N. events**	**IR (95% CI)**	**N. events**	**IR (95% CI)**	**N. events**	**IR (95% CI)**
CVEs	31	22.7 (15.4 – 32.1)	3	9.9 (2.1 – 28.9)	25	16.7 (10.8 – 24.6)
MACEs	9	6.6 (3.1 – 12.5)	0	-	9	6 (2.7 – 11.4)
TEs	8	5.9 (2.5 – 11.5)	2	6.6 (0.8 – 23.8)	3	2 (0.4 – 5.8)

List of abbreviations: *CVEs, cardio-cerebrovascular events; JAKi, Janus Kinase inhibitors; IR, incidence rate; CI, confidence interval; MACEs, major cardiovascular event; TEs, thromboembolic event.*

Table S6. Unadjusted and adjusted time-dependent Cox proportional hazard regression models on CVEs by the subgroups of JAKi exposure as reported in Figure 2.

	CVEs (n = 238)			
	Unadjusted		Adjusted	
**Parameter**	**HR (95%CI)**	**p**	**HR (95%CI)**	**p**
**Baricitinib vs TNFi**	1.41 (0.92 – 2.17)	0.119	1.17 (0.76 - 1.80)	0.479
**Tofacitinib vs TNFi**	0.62 (0.19 - 1.98)	0.418	0.52 (0.16 - 1.72)	0.283
**Upadacitinib/Filgotinib vs TNFi**	1.01 (0.63 - 1.60)	0.977	0.79 (0.49 - 1.26)	0.319
**IL-6i / RTX / ABT vs TNFi**	2.26 (1.67 – 3.07)	<0.001	1.42 (1.04 - 1.95)	0.028
**Age (years)**			1.04 (1.02 - 1.05)	<0.001
**eRHD-FI (per 0.1-point increment)**			1.81 (1.49 - 2.21)	<0.001
**Sex (M vs F)**			1.63 (1.24 - 2.15)	<0.001
**Previous treatment lines (1 vs 0)**			1.55 (1.16 - 2.08)	0.003
**Previous treatment lines (2 vs 0)**			1.05 (0.67 – 1.66)	0.826
**Previous treatment lines (≥3 vs 0)**			1.48 (0.92 - 2.39)	0.109
**NSAIDs (Y vs N)**			0.75 (0.57 - 0.99)	0.045
**GC (Y vs N)**			1.19 (0.91 - 1.57)	0.199
**Methotrexate (Y vs N)**			0.85 (0.65 - 1.11)	0.235
**CV drugs (Y vs N)**			2.33 (1.73 – 3.13)	<0.001

Abbreviations: *JAKi, Janus Kinase inhibitors; CVE, cardio-cerebrovascular event; HR, hazard ratio; CI, confidence interval; TNFi, Tumor Necrosis Factor inhibitors; IL6, Interleukin 6; RTX, Rituximab; ABT, Abatacept; e-RHD-FI, electronic-regional healthcare database frailty index; GCs, glucocorticoids; NSAIDs, not steroidal anti-inflammatory drugs; CV, cardio-cerebrovascular.*
